# Evaluation of Tissue Expression and Salivary Levels of HER2/neu in Patients with Head and Neck Squamous cell Carcinoma

**Published:** 2012

**Authors:** Soheil Pardis, Yasaman Sardari, Mohammad Javad Ashraf, Azadeh Andisheh Tadbir, Hooman Ebrahimi, Sara Purshahidi, Bijan Khademi, Mohammad Javad Fattahi, Marzieh Hamzavi

**Affiliations:** 1 *Department of Oral and MaxillofacialPathology, School of Dentistry, Shiraz University of Medical Sciences, Shiraz, Iran.*; 2 *Department of Pathology, School of Medicine, Shiraz University of Medical Sciences, Shiraz, Iran.*; 3 *Department of Oral Medicine, School of Dentistry, Shiraz University of Medical Sciences, Shiraz, Iran.*; 4 *Department of Otolaryngology, Khalili Hospital, Shiraz Institute for Cancer Research, Shiraz University of Medical Sciences, Shiraz, Iran.*; 5 *Shiraz Institute for Cancer Research, Shiraz University of Medical Sciences, Shiraz, Iran.*

**Keywords:** Carcinoma, HER2/neu, Salivary Gland, Squamous cell of head and neck, Tissue expression

## Abstract

**Introduction:**

HER2/neu, a member of the epidermal growth factor receptor family, has been shown to be over-expressed in some tumors. The purpose of this study was to determine the salivary levels and tissue expression of HER2/neu in patients with head and neck squamous cell carcinoma (HNSCC) and their correlation with clinicopathologic parameters.

**Materials and Methods:**

Enzyme-linked immunosorbent assays (ELISA) were used to evaluate the salivary levels of HER2/neu and immunohistochemistry was used to measure tissue expression of HER2/neu in 28 patients with HNSCC and 25 healthy control subjects.

**Results:**

The salivary levels of HER2/neu in patients with HNSCC were not significantly higher compared to healthy control subjects. There was no apparent correlation between salivary HER2/neu levels and clinicopathological features such as age, sex, tumor grade, tumor size and nodal status. All HNSCC specimens were positive (membranous or/and cytoplasmic) for HER2/neu, except one sample. Only one HNSCC specimen showed staining purely in the tumor-cell cytoplasm. All control specimens were also positive for both membranous and cytoplasmic HER2/neu but there was a significant difference between the level of cytoplasmic staining in the HNSCC specimens and in the control specimens (P<0.05).

**Conclusion:**

In our study, no overexpression of HER2/neu was observed. Thus, identification of HER2/neu levels plays no role in differentiating between normal and squamous cell carcinoma tissues or detecting the carcinogenesis process. Our findings suggest that the use of HER2/neu as a salivary marker of HNSCC is not recommended, because no significant preoperative elevation and no association with clinicopathological features were found.

## Introduction

Squamous cell carcinoma is the most common head and neck cancer (1), it is focally invasive and its behavior depends on the region where it originates. Each anatomic area has its own growth patterns and prognosis (2). Head and neck squamous cell carcinoma (HNSCC) has been a challenge to treat for a long time because of the high rates of recurrence and the advanced disease state usually present at the time of diagnosis. Molecular identification of tissue biomarkers in diagnostic biopsy specimens may not only identify patients at risk for developing HNSCC but may also identify patients who will benefit from more aggressive treatment modalities (3). 

The HER2/neu (ErbB) protein or epidermal growth factor receptors (EGFR) are a family of four structurally related receptor tyrosine kinases (4). The c-erbB-2 proto-oncogene (HER/NEU/neu) encodes a 185 transmembrane protein product of the tyrosine kinase family, with an extensive homology to EGFR, which has been mapped to region 21 of chromosome 17 (5), and can be activated by hetero-oligomerization with the other members of the ErbB family (6). Activation of the EGFR family (HER2/neu) by a variety of ligands is necessary for normal growth and differentiation (7). Increased levels of receptor ligands, co-expression of EGFR mutants, and cross-talk with HER2 or other receptors are mechanisms that can enhance EGFR signaling output and potentially alter the response to EGFR inhibitors (8). The dysregulation of these receptors is linked to multiple features of malignant tumors, including a loss of cell cycle control, resistance to apoptotic stimuli, invasiveness, chemo-resistance, and the induction of angiogenesis (9,10).

The use of targeted agents against molecular markers belonging to the EGFR family has recently become integrated into the treatment protocols of many malignancies, such as breast cancer (11-14). Despite recent improvements in the diagnosis and treatment of cancer, there are still many difficulties in evaluating the prognosis of head and neck carcinomas. Thus, recent reports have attempted to find more significant information to predict the biological behavior of this neoplasm, and to look at the possible relationship between tumoral progression and the products of genes that regulate cell proliferation and differentiation, such as the proto-oncogenes, anti-oncogenes and apoptosis-regulating genes. However, there is doubt over the prognostic significance of oncogene EGFR in these tumors, and thus its utility as a target of new therapies is still unclear (15-18). 

The aim of this study was primarily to examine the expression of HER2/neu in normal human oral epithelium and patients with HNSCC, to validate the controversial results of various studies and to determine whether or not HER2/neu could be considered as a useful marker for head and neck cancer. Thus, we investigated salivary levels of HER2/neu in healthy subjects and patients with HNSCC and also compared the tissue expression of the protein between the two groups.

## Materials and Methods

Study sample: In this study, 28 patients with HNSCC (22 males and 6 females, 58.0 ± 12.4 years of age) who had registered at Khalili and Chamran Hospital (affiliated to Shiraz University of Medical Sciences) and 25 healthy people (16 males and 9 females, 56.9 ± 13.4 years of age) were evaluated. The case and control groups were matched by age and sex. Patients and control subjects who showed signs of significant morbidity, active medical problems, and any systemic or inflammatory disease were excluded from the study. Patients with a histopathological diagnosis of HNSCC as well as a large enough tissue sample with H & E stained slides that could be evaluated, and 25 control subjects with normal oral epithelial tissue were enrolled in the research. Clinical data, such as age, gender, location of the tumor, TNM, and tobacco consumption, were obtained from medical records. The Ethical Committee of Shiraz University of Medical Sciences approved the study. All subjects were informed about the research and agreed to participate in the study by signing an informed consent form. 

Saliva collection and analysis: Salivary samples were taken from subjects before any surgical procedures or chemotherapy protocols commenced. Prior to the collection of unstimulated whole saliva, subjects were asked to refrain from eating, drinking, smoking or performing oral hygiene procedures for 30 minutes. The lip area was cleaned and each subject rinsed their mouth once with plain water. Typically, patients donated 5 to 10 ml of saliva. Samples were then centrifuged at 2,600×g for 15 minutes at 4ºC. The resulting supernatant was then stored at -80ºC until use (19). Salivary protein levels were measured by sandwich ELISA, in accordance with the procedures recommended by the manufacturer (BMS 207: Bender Med System GmbH, Germany 4).

Immunohistochemical analysis: Sections of tumor tissue (4 µm thick) were mounted on positively charged microscope slides. After dewaxing in xylene, sections were dehydrated in ethanol and rinsed in distilled water. Antigen retrieval was performed using DAKO Target Retrieval Solution (DAKO, Carpinteria, CA). The endogenous peroxidase was quenched with 3% H2O2. Peroxidase-labeled polymer conjugated to goat anti-mouse HER2 was then used to detect the antigen-antibody reaction (DAKO EnVision System; DAKO Corporation, Carpinteria, CA). The HER2 antibodies (ErbB 2 antibody ab2428, DAKO Corporation, Denmark; 1:200 dilution) were incubated with the tissue sections for 1 hour at room temperature. Sections were then visualized with 3,3-diaminobenzidine as a chromogen for 5 minutes and counterstained with Harris’s hematoxylin. Slides were washed in tap water, dehydrated, and mounted with glass coverslips. Positive controls were sections of breast cancer tissue that had previously been found to be positive for HER2/neu and negative controls consisted of duplicated sections of the same specimens in which the primary antibody had been excluded and replaced with PBS.

In order to quantify the expression of HER2/neu, representative tumor sections were identified on a light microscope. Ten random fields were chosen for each section and the total numbers of positive cells for all 10 examined fields were counted and the percentage of staining was calculated (7). Membranous and/or cytoplasmic staining was regarded as positive. The percentage of membranous and/or cytoplasmic stained tumor cells was calculated separately for each section. Tumors with more than 1% of cells stained for HER2/neu were considered positive. The percentage of positivity ranged from 1% to 100% (20,21). The immunostaining was analyzed using a score system, which considered the proportion of tumor cells with membranous and/or cytoplasmic staining as 0 for negative; 1 for less than 33%; 2 for 33% to 66%; and 3 for more than 66% and the intensity of immunostaining as 0, 1+, 2+, or 3+. The results obtained were multiplied together, yielding a cytoplasmic/membranous immune-staining grade scale, with steps of 0, 1+, 2+, 3+, 4+, 6+, and 9+, where 0 was considered to be negative staining, 1+ and 2+ slight staining, 3+ and 4+ moderate staining, and 6+ and 9+ intense staining. All immune-staining assessments were blinded to the clinical data. All slides were observed by two pathologists, separately, who scored the immunostaining twice to decrease inter-observer variability, in a blinded fashion. In unmatched cases, slides were evaluated again by both pathologists using a multi-headed microscope.

**Fig 1:**
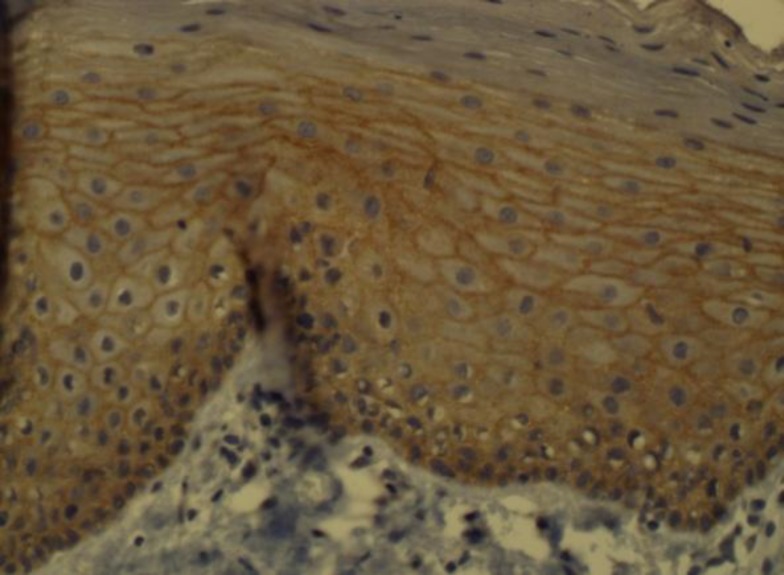
Cytoplasmic staining in the basal and parabasal layers of normal epithelium

Statistical analysis: The Mann-Whitney test, Student’s independent t-test, Chi-Squared test and Fisher’s exact test were used for statistical analysis. The level of significance was set at 0.05.

## Results

The clinical data for the patients with HNSCC included in this study of HER2/neu expression are presented in (Table 1).

**Table 1:** Clinico-pathological profile of 28 patients with head & neck squamous cell carcinoma

**Age (years)**	**58.0 ± 12.3**
**Gender**	
Male	19 (67.8%)
Female	9 (32.2%)
	
**Tumor size**	
T1	0 (0%)
T2	28 (100%)
T3	0 (0%)
	
**Regional lymph node involvement**	
N0	24 (85.7%)
N1	4 (14.3%)
N2	0 (0%)
N3	0 (0%)
**Distant metastasis**	
M0	28 (100%)
M1	0 (0%)
	
**TNM stage**	
I	0 (0%)
II	24 (85.7%)
III	4 (14.3%)
IV	0 (0%)
**Histological grade**	
I (well differentiated)	8 (28.6%)
II (moderately differentiated)	17 (60.7%)
III (poorly differentiated)	3 (10.7%)
Larynx	22 (78.5%)
Oral cavity	6 (21.5%)

All samples of normal oral epithelium were positive for membranous and cytoplasmic HER2/neu. Cytoplasmic staining was limited to the basal and parabasal layers in normal epithelium (Figure 1).

**Fig 2:**
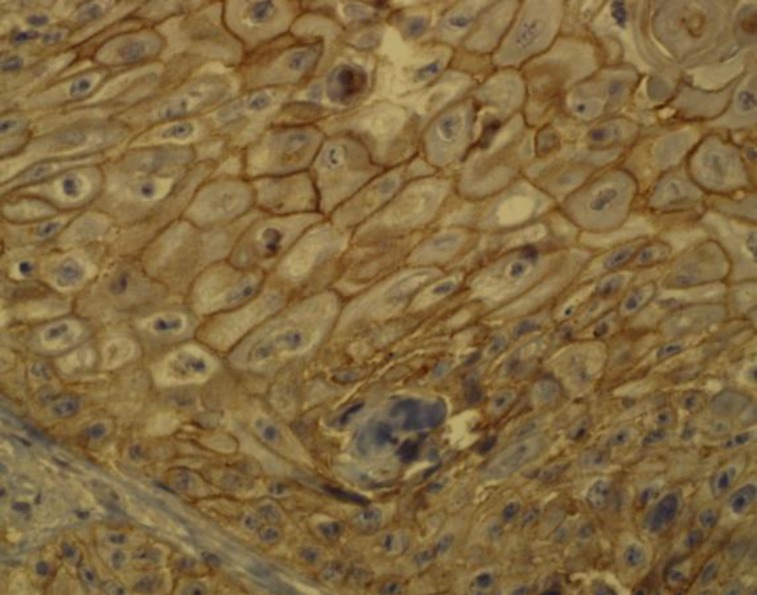
Membranous staining in squamous cell carcinoma tissue (×400

**Fig3:**
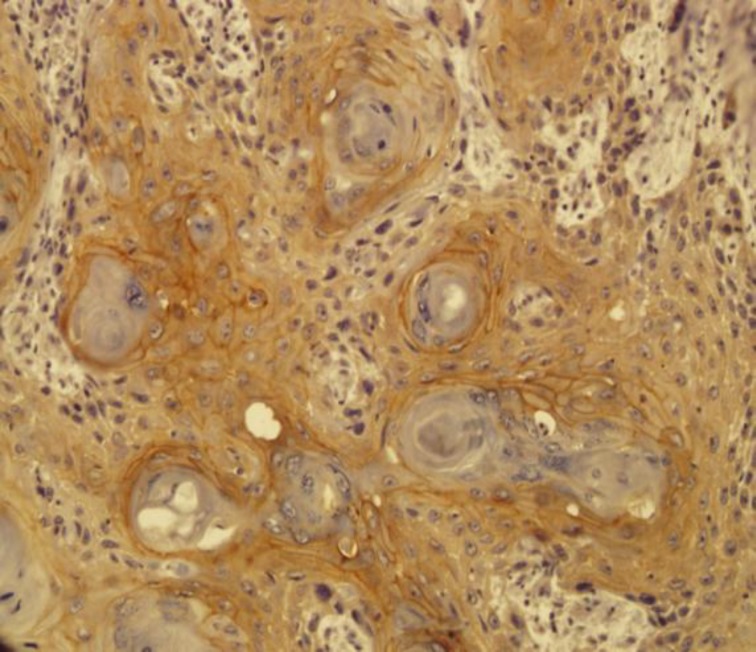
Cytoplasmic and membranous staining in squamous cell carcinoma tissue

All HNSCC specimens were positive for membranous or/and cytoplasmic HER2/neu, except one sample (Figure 2,3).

Only one HNSCC specimen contained only cytoplasmic staining. The mean percentage of membranous staining in HNSCC samples was 63.0 ± 30.8% and in normal epithelium it was 66.4 ± 16.4%. There was no significant difference in the percentage of membranous staining between the patient and control groups. 

**Fig 4:**
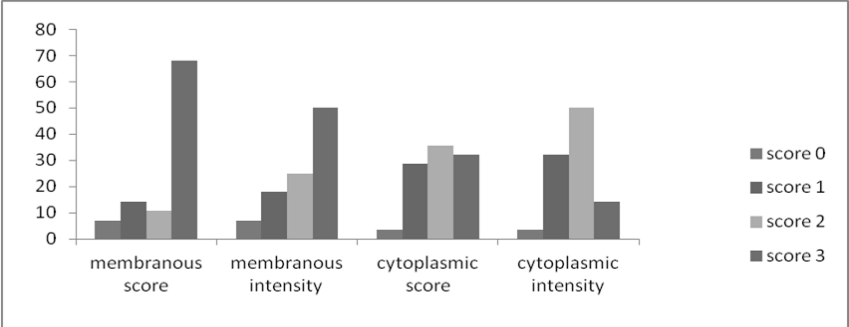
Distribution of score and intensity of membranous and cytoplasmic HER-2/neu immunohistochemical staining in HNSCC specimens

The distribution of membranous immune-staining according to each of the grades is presented in (Figure 4). 

**Fig 5:**
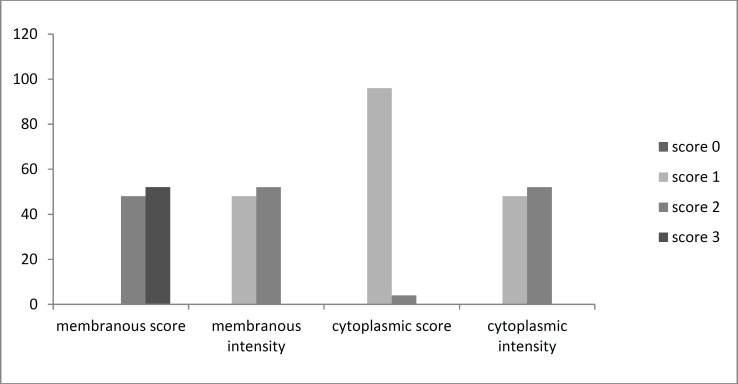
Distribution of score and intensity of membranous and cytoplasmic HER-2/neu immunohistochemical staining in control specimens

There was no significant difference between the grade of membranous immune-staining in the case and control groups. However, the mean percentage of cytoplasmic staining in samples of HNSCC was 48.6 ± 29.1% and in normal epithelium it was 13.2 ± 6.7%. There was a significant difference in the percentage of cytoplasmic staining between the case and control groups (*P*<0.001).The distribution of cytoplasmic immune-staining grades is presented in (Figure 5).

There was a significant difference between the grade of cytoplasmic immune-staining in the case and control groups (*P*<0.001). There was no correlation between the percentage of HER-2 staining and any clinicopathologic factors.There was a high tendency for cytoplasmic staining around keratin pearls in well-differentiated HNSCC samples.

The mean salivary level of HER2/neu in patients with HNSCC was 3.12 ± 4.58 ng/ml and for the control group was 13.2± 6.75 ng/ml. There was no significant difference in salivary concentrations of HER2 between the case and the control groups. There was also no significant correlation of salivary levels with the percentage of overall immune-staining in samples of tumor tissue from patients with HNSCC, and there was no significant correlation between salivary HER-2 levels and the clinic-pathological data of patients. However, salivary HER-2 levels had a reverse relationship with the percentage of cytoplasmic staining in tumor samples from patients with HNSCC, but there was no significant correlation(co.co.= -0.025, *P*= 0.90).

## Discussion

We studied HER2/neu expression in patients with HNSCC and its correlation with salivary levels of this marker. Although a lack of specificity of tumor markers and a lack of sensitivity of testing systems has been noted, which limits their clinical use, finding biomarkers for cancers would allow physicians to identify individuals who are susceptible to certain types and stages of cancer. It would also allow the development of tailored preventive and therapeutic modalities based on a patient’s genotype and phenotype information. These biomarkers should be cancer specific and detectable at a high level of sensitivity in a wide range of specimens containing cancer-derived materials, including body fluids (plasma, serum, urine, saliva, etc.), tissue samples, and cell lines (5).

The amplification of the HER2 gene has been demonstrated in many carcinomas of glandular origin, and immunohistochemically-identified expression of the gene has been proven to be closely associated with its amplification level (21). The amplification and consequent over-expression of the HER2 gene, as well as its relationship with tumorogenesis was first reported by Schechler et al. (1985) in neuroglioblastomas in rats (22). The dysregulation of these receptors is linked to multiple features of malignant tumors, including a loss of cell cycle control, resistance to apoptotic stimuli, invasiveness, chemo-resistance, and the induction of angiogenesis (10).

Reports on the role of the HER2/neu proto-oncogene product in HNSCC are less conclusive than those detailing the role of EGFR(HER1), as over-expression of HER2/neu has been shown to be present in anywhere from only a few to all HNSCC specimens investigated and correlations with clinical parameters are controversial (23-26). In our study, all samples except one expressed HER2/neu and there was no significant difference between the overall level of staining in the case and control specimens. All non-tumoral and 96.4% of HNSCC samples were positive for cytoplasmic and/or membranous HER2/neu. One of the HNSCC specimens was positive only for cytoplasmic HER2/neu and another one was negative for the marker. Thus, we found no over-expression of HER2/neu in HNSCC tissues. This implies that abnormal expression and over-expression of HER2/neu do not play a role in the carcinogenesis of HNSCC. This result is in accordance with several other studies that found no HER2/neu over-expression in cases of HNSCC (26-29).

On the other hand, some studies have reported over-expression of HER2/neu as a potential useful marker in distinguishing non-cancerous from cancerous tissues (14,30). Fong et al. suggested that there are dynamic changes in HER2/neu expression during the process of carcinogenesis in head and neck cancer (31). Wilkman et al.in 1998, reported an increase in HER2/neu expression during the progession from normal mucosa to hyperkeratosis and to dysplasia and HNSCC (32). In our study there was no difference in the percentage of membranous staining of HER2/neu in the case and control groups but there was a significant difference in cytoplasmic staining between the case and control groups. It should be mentioned that cytoplasmic staining in the normal epithelial specimens was specific to the basal and parabasal layers. 

In various studies purely membranous (25) or cytoplasmic (25,26), and mixed membranous-cytoplasmic (15,23,25,33) expression have been reported. In squamous cell carcinomas, cytoplasmic staining has been widely reported; however, its interpretation is not clear at present. It has been argued that cytoplasmic staining may be a technical artifact due to cross-reactivity of antibodies, possibly with keratin or antigen retrieval (14). Others, however, propose that it may represent true protein over-expression that is probably due to incomplete receptor degradation (34). Some antibodies, such as CB11, have a tendency towards producing cytoplasmic staining, thus some manufacturers suggest that purely cytoplasmically stained samples should be designated as negative (35). However, the importance of cytoplasmic staining and whether or not it may be evaluated when determining HER-2 expression in OSCC/ HNSCC is controversial. 

In our study we evaluated cytoplasmic and membranous staining separately to make it clear and found a significant difference between cytoplasmic immune-reactivity in the case and control groups. In the literature, the percentage of HER-2/neu positivity in patients with HNSCC is extremely variable. It is possible that these discrepancies in results may be attributed to an initial lack of standardization of the assay methods (14). Another reason for controversial results in different studies might be due to use of different immunohistochemical methods (direct, indirect), type of antibody (clone CerbB2, CB11, ICR1b, polyclonal DAKO, monoclonal zymed), no specific criteria for positive staining of HER2/neu protein (membrane and/ or cytoplasmic) and/or using different techniques (immunosorbent assay, radioimmunoassay, IHC) or the different locations of the lesions and sex of patients with HNSCC. 

There was no significant correlation between HER2/neu expression and age, gender, tumor size, lymph node and distant metastasis, tumoral stage and histologic differentiation in our study. In 1997 Xia et al. found a strong correlation between cerbb2 over-expression and overall survival of patients with OSCC (34). In a review study in 2001, Quon et al. indicated that high expression levels of EGFR and HER2/neu are prognostic markers which correlate with poor clinical outcome in patients with HNSCC (36).By contrast, in a case review study in 2009 by Tse et al. HER2 was associated with longer survival in node-positive patients (37). Some studies had evaluated the correlation of clinicopathologic data such as tumor stage with HER2/neu expression. In 2008, Fong et al. found that HER2/neu expression was significantly higher in patients with advanced stage IV tumors than in those with stage I–III tumors (31).

One of the limitations of our study was the lack of stage IV tumors in the patients with HNSCC, thus we could not fully judge the relationship between level of tumor stage and HER2/neu expression. In our study, there was prominent cytoplasmic staining around keratin pearls in well-differentiated samples but there was no significant correlation between HER2/neu cytoplasmic staining and tumor grade. In recent years, increasing interest has developed in the use of saliva as an adjunct test medium to help in conventional medical assessment of serious systemic diseases (38). Due to the simplicity of collection, saliva may be collected repeatedly with minimal discomfort to the patient. This noninvasive process thereby renders saliva a very desirable diagnostic medium. More importantly, saliva contains constituents that are frequently altered in the presence of systemic diseases (39). Because of these significant characteristics, finding biomarkers in saliva for the detection of serious systemic illnesses, such as cancer, is of great interest to most salivary researchers.

In our study, there was no significant difference in salivary levels of HER2/neu between the HNSCC and control groups. The salivary level of HER2/neu in patients with HNSCC was lower than in the control group but the difference was not significant. Also, there was an inverse relationship between salivary levels of HER2/neu and the percentage of cytoplasmic staining. One of the reasons for the presence of lower HER2/neu salivary levels in the case group could be the high level of cytoplasmic staining of this marker in HNSCC specimens, which could cause decreased membranous transportation of this marker to saliva. The inverse relationship between salivary levels of HER2/neu and cytoplasmic staining is proof for this claim.

In this study there was no significant correlation between the salivary levels of HER2/neu and the clinicopathological data of the patients. Briefly, we found that there was no increase in the presence of HER2/neu protein in saliva in patients with more progressive or aggressive lesions. In a similar study in 2010, Vanessa et al. also found no significant association between the salivary levels of the proteins and patient clinicopathological data, such as age, tumor site, histological grading, T status, nodal involvement of the tumor or stage. Salivary levels of HER-2 also showed no difference between patients pre-surgery and healthy control groups; however, both demonstrated an increase after surgical removal of the tumor (19).

In our cases, no overexpression of HER2/neu was observed. Thus, the protein cannot be used to differentiate between normal and squamous cell carcinoma tissues or identify the occurrence of carcinogenesis.

## Conclusion

Our findings suggest that the use of HER2 as a salivary marker of HNSCC is not recommended because no significant preoperative elevation of HER2 or association with clinicopathological features was found. 
